# Facial Regulation During Dyadic Interaction: Interpersonal Effects on Cooperation

**DOI:** 10.1007/s42761-023-00208-y

**Published:** 2023-08-23

**Authors:** Danielle Shore, Olly Robertson, Ginette Lafit, Brian Parkinson

**Affiliations:** 1 Department of Experimental Psychology, University of Oxford, New Radcliffe House, Oxford, OX6 2RR UK; 2 Department of Psychiatry, University of Oxford, Oxford, UK; 3 Research Group for Quantitative Psychology and Individual Differences, KU Leuven, Leuven, Belgium; 4 Center for Contextual Psychiatry, Department of Neuroscience, KU Leuven, Leuven, Belgium

**Keywords:** Cooperation, Emotion regulation, Facial signals, Social influence

## Abstract

This study investigated interpersonal effects of regulating naturalistic facial signals on cooperation during an iterative Prisoner’s Dilemma (IPD) game. Fifty pairs of participants played ten IPD rounds across a video link then reported on their own and their partner’s expressed emotion and facial regulation in a video-cued recall (VCR) procedure. iMotions software allowed us to auto-code actors’ and partners’ facial activity following the outcome of each round. We used two-level mixed effects logistic regression to assess over-time actor and partner effects of auto-coded facial activity, self-reported facial regulation, and perceptions of the partner’s facial regulation on the actor’s subsequent cooperation. Actors were significantly less likely to cooperate when their partners had defected on the previous round. None of the lagged scores based on auto-coded facial activity were significant predictors of cooperation. However, VCR variables representing partner’s positive regulation of expressions and actor’s perception of partner’s positive regulation both significantly increased the probability of subsequent actor cooperation after controlling for prior defection. These results offer preliminary evidence about interpersonal effects of facial regulation in interactive contexts and illustrate how dynamic dyadic emotional processes can be systematically investigated in controlled settings.

**Supplementary Information:**

The online version contains supplementary material available at 10.1007/s42761-023-00208-y.

Is that your best offer or should I hold out for more? Will you take advantage if I disclose any weakness? Many situations require us to anticipate someone else’s behavior before deciding how to act. Knowing that others do not always do what we want, we look for clues about their true intentions. In direct interactions, many of these clues come from faces. For example, smiles often signal affiliative motives (e.g., Scharlemann et al., 2001) whereas scowls imply antagonism (e.g., Yik & Russell, 1999). But facial signals can also mislead us. People may exaggerate, suppress, or mask expressions to conceal their intentions or convey misinformation (e.g., Ekman, 1972). The present research used an innovative combination of methods to investigate facial regulation in an ongoing interpersonal interaction involving both cooperative and competitive motives. Specifically, we assessed whether one interactant’s suppression or exaggeration of facial signals influenced the other interactant’s choices, and whether that other interactant’s perception of their partner’s regulatory attempts reduced subsequent cooperation.

## Interpersonal Effects of Facial Signals

It is widely acknowledged that facial signals can affect other people’s behaviour (e.g., Crivelli & Fridlund, 2018; Parkinson, 2019). Several studies have investigated these effects in controlled interpersonal interactions, often focusing on smiles. For example, Scharlemann et al. (2001) showed that participants allocated more funds to trust-game players whose picture showed them smiling (see also Xiong et al., 2021), and Centorrino et al. (2015) found similar effects of prerecorded videos of smiles perceived as genuine.

Instead of presenting separately prepared facial stimuli, a few studies have assessed interpersonal effects of naturalistic facial activity. For example, Reed et al. (2012) coded facial behavior during an “acquaintance” period before a one-shot Prisoner’s Dilemma game. Participants cooperated less with players who had expressed an intention to cooperate but shown more negative expressions. Danvers and Shiota (2018) found that players whose prior smiling was dynamically dependent on their partner’s smiling solicited greater cooperation. Similarly, Deng et al. (2022) showed that Duchenne smiles and direct gaze predicted subsequent cooperation.

These studies consistently suggest that smiles can encourage trust and cooperation from others whereas negative facial signals can inhibit them. However, in all cases, the outcome variable was a one-shot decision assessed in a separate task. Different processes may operate when facial signals are contextually relevant to unfolding behavior. To investigate such processes, de Melo et al. (2014) programmed an avatar either to show smiles following mutual cooperation and regret following unreciprocated cooperation in an IPD game or to show the opposite response pattern. Participants cooperated more with the first avatar although cross-condition levels of smiling were equivalent. Inferred appraisals mediated the effect, suggesting that context-dependent signals communicate the signaller’s changing orientation.

However, facial signals can conceal as well as reveal intentions. Correspondingly, perceivers may not respond directly to the apparent meanings of facial signals when they suspect deception. For example, Côté et al. (2013) found that bargainers pulling angry faces solicited less favourable deals than those who worked up genuine anger, and Shore and Parkinson (2018) found that suspected regulation reduced the positive impact of verbally expressed guilt following betrayal in a trust game. Thus, expressive regulation may backfire when detected by others. The present research assessed effects of facial regulation on cooperation in an interactive task where signals are calibrated with ongoing game-events.

### The Present Study

Although some studies have measured dynamic dyadic signals (e.g., Danvers & Shiota, 2018; Deng et al., 2022), they have not usually assessed interpersonal effects of facial activity produced during ongoing interactions where both parties are attempting to anticipate each other’s next move. Such a setting provides more direct evidence of strategically regulated facial activity and its real-time interpersonal perception. Our study therefore assessed two-way facial communication throughout an interactive IPD game. Because our focus was on facial signals, we disabled sound on the video channel linking participants. We used software to auto-code facial muscle movements time-linked to simultaneously experienced game-events and VCR (Gottman & Levenson, 1985) to collect participants’ reports of their own and their partner’s expressed emotions and facial regulation.

Our previous regression-based analyses of the present dataset found weak correspondence between self-rated, partner-rated, and auto-coded facial measures (Hoegen et al., 2019). The analyses reported here used a more sophisticated over-time actor-partner interdependence model (APIM; Kenny et al., 2006) to determine whether partner’s prior facial behavior and facial regulation, and actor’s perceptions of these variables, influenced participants’ subsequent game decisions. This allowed us to assess whether documented effects of regulated expressions (e.g., Côté et al., 2013) apply when facial movements are unmanipulated and genuinely interactive.

The analyses reported in this paper were preregistered after all study data had been collected. The hypotheses were that prior cooperation by actor and partner would encourage the actor’s subsequent cooperation (hypothesis 1), but that prior auto-coded and VCR facial valence (and its perception by the partner) would further increase cooperation (hypothesis 2). We also predicted significant effects of partner’s prior facial regulation (hypotheses 3) and actor’s perceptions of it (hypothesis 4). Further, we assessed whether auto-coded Duchenne smiles and three other facial configurations would predict cooperation after controlling for prior actor and partner cooperation (hypothesis 5; see https://osf.io/dzrc3/ for all hypotheses).

## Method

### Ethical Approval

All procedures were approved by Oxford Tropical Research Ethics Committee (OxTREC). The reference number for the ethics application was 513-17.

### Participants

We recruited 100 participants (61 females and 29 males, mean age = 26.42, *SD* = 7.44, see Table 1 for demographic information) from a community panel (69 participants) and using local advertisements (31 participants). These two samples did not differ significantly in age, gender, or ethnicity.^1^ All participants provided written informed consent, but one withdrew consent after participating and the data from the dyad including that participant were consequently removed from analysis. Three additional dyads were excluded only from those analyses that used video-cued recall data, one because the computer failed to capture the full set of scores for one dyad member and two because of video playback issues. Seven dyads including participants with at least 20% missing iMotions facial expression data (e.g., those whose faces had moved out of the camera frame) were excluded only from those analyses that used scores based on auto-coded facial activity (in line with prior research, Kulke et al., 2020).^2^ In sum, we maximised the sample size for each analysis based on the data available in each case, meaning that the Ns reported below differ depending on the results that are reported. Each participant was paid £10 for participation and earned chances to win a first prize of £100 and two second prizes of £50 by acquiring more points during the IPD game (see below). We ran a sensitivity power analysis to determine the minimum detectable effect size if the study were replicated using procedures developed by Lafit et al. (2022). For a population similar to that sampled in the present study, this analysis showed that a replication study using 45 dyads and 10 rounds would yield greater than 90% power if the effect of participant’s own valence on the decision to defect was smaller than .95 (see Supplementary Materials for further details).

**Table 1:** Participant demographic data (*N*_Person_ = 90, *N*_Dyad_ = 45)

	Mean	*SD*	Median	Min	Max
Age	26.64	7.57	24	18	59
		Observations		Percentage	
Gender					
*Female*		61		67.8%	
*Male*		29		32.2%	
Ethnicity					
*Caucasian*		69		76.7%	
*Mixed/multiple ethnic groups*		5		5.6%	
*Asian/Asian British*		8		8.9%	
*Black/African/Caribbean/Black British*		3		3.3%	
*Other ethnic groups*		5		5.6%	

### Procedure

**Fig. 1:**
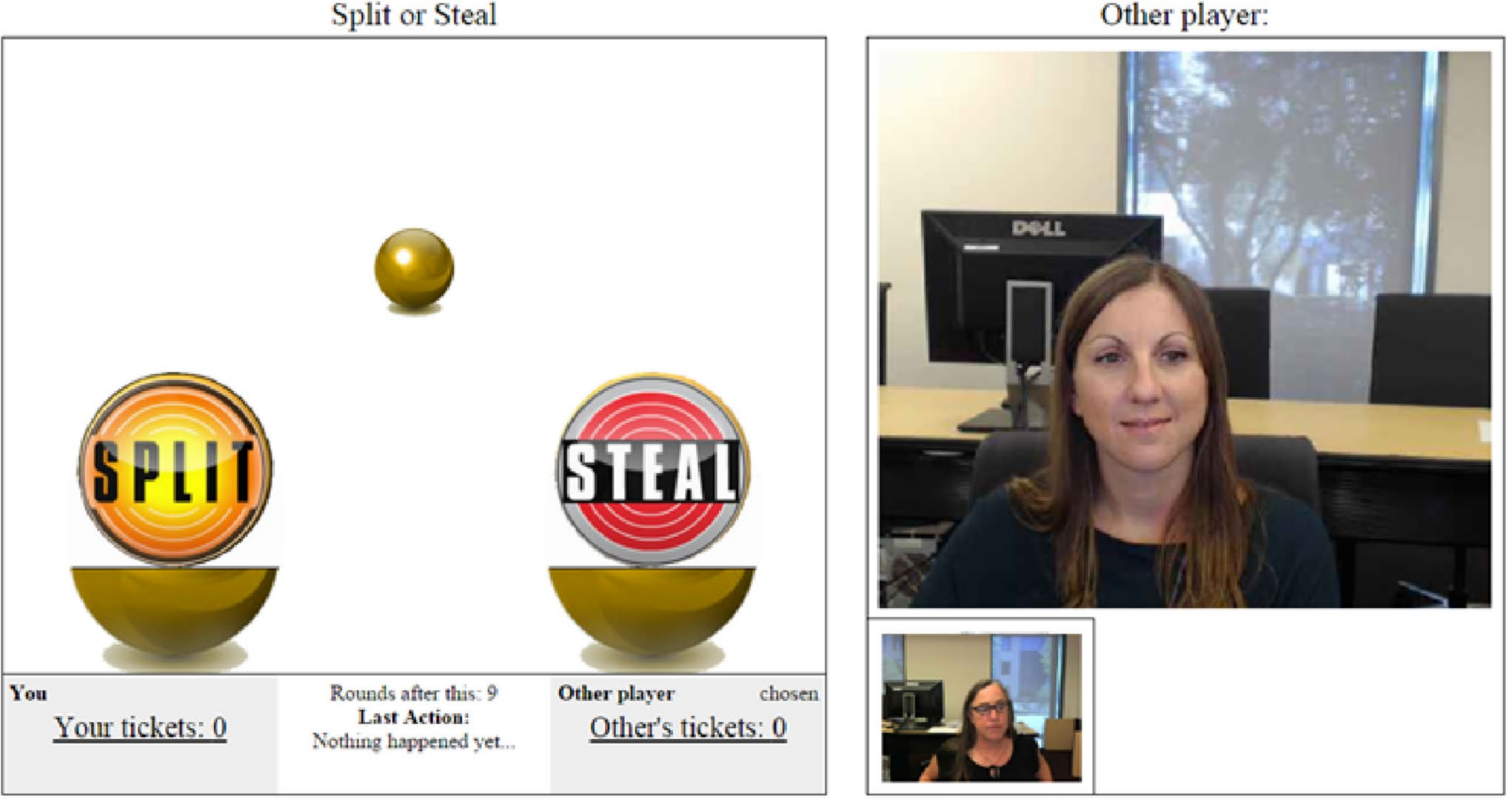
Screen display during decision phase of Prisoner’s Dilemma game. Participants selected either the “split” or “steal” ball on each round. Participants could see the number of tickets won by both players as well as the other player’s live video feed (big window) and their own video feed (small window) throughout the game. Image taken from Hoegen et al., 2015

Randomly paired participants played 10 IPD rounds with each dyad member in a separate cubicle connected to the other dyad member’s cubicle by a video link with the sound channel disabled. The computer monitors in each cubicle showed a real-time presentation of both participants’ faces and the current state of the game (see Fig. 1). Participants were told that they should not talk or use hand gestures during the procedure. Game events were programmed by Hoegen et al. (2015) who based the graphics and decision labels on the UK TV show *Golden Balls*, which was originally broadcast between 2007 and 2009. In each round, participants played for a set of lottery tickets and selected whether to “Split” (cooperate) or “Steal” (defect) by clicking the relevant button on the screen. Once both participants had made their choices, the outcome of the round was displayed to both participants.

The outcome for both players depended on their joint decisions, with maximum joint profit for matched co-operation (CC) and minimum joint profit for matched defection (DD). In mixed outcomes (CD and DC), the individual profit for the defector exceeded the individual profit after joint cooperation (see Table 2 for the payoff matrix). Participants were informed that each ticket that they earned would be entered into a lottery draw for a monetary prize and that they could therefore increase their chances of winning a prize by collecting more tickets. A ticket counter displayed on the screen allowed participants to track their current scores.

**Table 2:** Payoff matrix for levels of cooperation and defection between dyad members

		Participant B	
		Cooperate (split)	Defect (steal)
Participant A	Cooperate (split)	A = 5, B = 5	A = 0, B = 10
	Defect (steal)	A = 10, B = 0	A = 1, B = 1

After the game, participants completed a video-cued recall (VCR) procedure, in which they made ratings of the valence of both their own and their partner’s expressions and the extent to which they and their partner were regulating positive and negative expressions. By using the specific timing of participants’ decisions as logged in the database, we could automatically generate video clips of specific events. For every participant, we created 5-second (150-frame) video clips starting immediately after information about both players’ decisions and the outcome of each round was presented (*outcome clips*).^3^

After the VCR procedure, participants answered questions assessing their general game experience and impressions of their game partner (see Supplementary Materials). Finally, participants were debriefed and thanked for their participation.

### VCR Measures

Participants individually viewed their outcome clips in chronological order and provided ratings based on each of them. They first rated the video of themselves and then the video of their opponent for each clip. For each presented 5-second video clip, they rated the valence of their expressed emotion and the extent to which they had regulated positive and negative expressions at the time. Participants also responded to corresponding items about their partner’s emotions and facial regulation. The valence item was “How positive/negative was the emotion that you were [your partner was] expressing?” rated on a slider running from −50 (very negative) to +50 (very positive). The positive regulation item asked “To what extent were you [was your partner] regulating your [their] expression of positive emotions?” and the negative regulation item simply replaced the word “positive” with “negative.” The rating slider for the two regulation items ran from −50 (suppressed) to +50 (exaggerated).

### Post-task Questionnaire Measures

A final set of measures were presented in an on-line questionnaire administered after the VCR procedure was complete. We do not report analyses of these measures in this paper, but the full set of questionnaire measures is provided in Supplementary Materials.

### Auto-coding of Facial Activity

Facial configurations from the webcam videos were auto-coded using the AFFDEX by Affectiva module of iMotions software (McDuff et al., 2017). AFFDEX outputs frame-by-frame intensity values for 20 observable muscle movements or “action units” (AUs), as defined by the Facial Action Coding System (FACS; Ekman & Friesen, 1978). AFFDEX has been found to reliably detect and report AU activation, with Receiver Operator Characteristic scores ranging from .75 to .96 (McDuff et al., 2017). Whilst validation is predominantly based on static images, recent research suggests that AFFDEX performance is comparable to facial EMG when identifying happy and angry expressions from videos (Kulke et al., 2020). AFFDEX codings of joy from 1-minute clips and 1-second slices taken from videoed clinical interviews have also been found to be consistent with those produced by a different auto-coding system (FaceReader 6.0, Noldus, 2014; ICC = .86) and by trained human coders (ICC = .73), although codings of other expressions diverged (Gupta et al., 2022). However, non-standard expressions are sometimes misclassified as neutral by both human coders and automatic recognition software (Küntzler et al., 2021). Additionally, auto-coding accuracy is more affected by image quality (e.g., lighting) than is the accuracy of human coding (Cross et al., 2023), so we took care to ensure adequate lighting for videos recorded in this study.

AU activation values range from 0 to 100, where 0 represents no activity and 100 indicates full activation. The outcome clips were analysed for each of the 10 trials completed by each participant. To facilitate direct comparison with our emotion and regulation ratings from the VCR measures, we focused mainly on valence scores ranging from −100 (negative) to +100 (positive) as computed by Affectiva. Valence is calculated based on combined intensity scores for a set of action units thought to be associated with positive emotions (AU6 Lip Corner Puller, AU12 Cheek Raiser) and negative emotions (AU1 Inner Brow Raiser, AU4 Brow Furrow, AU9 Nose Wrinkle, AU10 Upper Lip Raiser, AU15 Lip Corner Depressor, AU17 Chin Raiser, AU24 Lip Presser, AU26 Lip Suck) with the former increasing the positivity of scores and the latter increasing the negativity of scores. We also calculated scores for three distinct and consistent configurations of facial muscle movement identified from our previous analyses of video data collected during IPD games (see Robertson et al., 2023). These configurations were “reward smiles” (AU12 Lip Corner Puller, AU27 Mouth Open, and AU6 Cheek Raiser), “mouth-based appeasement” (AU14 Dimpler, AU17 Chin Raiser, AU24 Lip Pressor, AU20 Lip Stretch and AU28 Lip Suck), and “brow and upper lip disapproval” (AU4 Brow Furrow, AU9 Nose Wrinkler and AU10 Upper Lip Raiser). Finally, we coded the combination of AU6 and AU12 to index Duchenne smiles which have previously been shown to predict cooperation (e.g., Centorrino et al., 2015).

For the lagged analyses, we analysed 5-second clips of the webcam videos taken immediately following the reveal of gameplay decision (facial responses to the outcome of the round). We calculated the mean of the AFFDEX scores across the 150 frames of the outcome clips (sampled at 30fps). We also calculated mean scores for each participant across all 10 outcome clips so that we could assess between-subjects correlations with aggregated scores of other variables.

### Design

The game outcomes experienced by each participant were dependent on the decisions of both members of the pair. Similarly, each participant’s facial behavior responded to and operated upon the facial behavior presented by the pair’s other member. The study therefore had a dyadic design. Further, decisions on each trial were partly dependent on the outcomes of previous trials, meaning that analyses needed to assess sequential effects over time. The main dependent variable was a participant’s decision to cooperate (split) or defect (steal) on each trial (a binary categorical variable), and the predictors were time-lagged actor and partner cooperation decisions, time-lagged auto-coded facial signals, and time-lagged self and partner ratings of facial regulation collected during the VCR procedure.

### Analysis Strategy

In our dataset, repeated measurements from the 10 rounds of IPD were nested within indistinguishable partners in each dyad. To analyse data with this structure, we used two-level mixed-effects models estimated using an over-time (longitudinal) Actor-Partner Interdependence Model (APIM; Kenny et al., 2006). We used a mixed-effects logistic regression to predict cooperation where the outcome was a dummy variable representing the actor’s decision (0 = cooperate, 1 = defect) at round *t*, and the predictors were *t* − 1 (lagged) values of the hypothesised predictor variables. A significant *actor effect* meant that the participant’s own score from the previous round reliably predicted their own subsequent cooperation and a significant *partner effect* meant that the participant’s partner’s score from the previous round reliably predicted the actor’s subsequent cooperation. Because partners were treated as indistinguishable, the sizes of actor and partner effects do not differ across partners overall. To account for differences across dyads, we introduced a random effect for the intercept. We person-mean centred all predictors except the dummy variables representing prior decisions to obtain fixed estimates that reflect their (average) within-dyad associations with the outcome. A *z*-test with a *p*-value of .05 was used to assess fixed effects. All multilevel models were estimated using the R package lme4 (Bates et al., 2015).

## Results

### Descriptive Statistics

Participants cooperated in 74.56% of trials, with mutual cooperation in 60.67% of trials. Mutual defection happened in 11.56% of trials, leaving 27.78% of trials where one member of the pair cooperated and the other defected. 16 dyads cooperated across all rounds, meaning defection occurred at least once during gameplay in 33 dyads. Descriptive statistics for other key variables are presented in Table 3.

**Table 3:** Descriptive statistics (*N*_Person_ = 90, *N*_Dyad_ = 45).

Variable	Mean (*SD*)	*SD*	Median	Min	Max
Decision (% defect)	25.44				
Mutual cooperation (%)	60.67				
Mutual defection (%)	11.56				
Valence (AC)	15.64	30.76	1.10	−93.77	99.63
Valence (VCR)	8.75	17.12	6	−50	50
Positive regulation (VCR)	−3.53	15.49	0	−50	50
Negative regulation (VCR)	−3.61	12.05	0	−50	50
Perceived partner valence (VCR)	8.78	16.20	6	−46	50
Perceived partner positive regulation (VCR)	−2.16	15.27	0	−50	44
Perceived partner negative regulation (VCR)	−4.32	11.66	0	−50	50
Duchenne smile (AC)	28.12	32.33	12.64	0	100

*AC* auto-coded, *VCR* video-cued recall

### Between-Participants Correlations

We computed aggregated scores across trials for each participant based on VCR ratings of expression and regulation and averaged auto-coded facial data for all outcome clips. Table 4 presents correlations between these aggregated scores and overall cooperation scores based on the total number of trials in which each participant cooperated.

**Table 4:** Correlations between aggregated predictors and post-game outcomes (*N* = 82^+^)

	Valence (AC)	Partner valence (AC)	Valence (VCR)	Perceived partner valence (VCR)	Positive regulation (VCR)	Negative regulation (VCR)	Perceived partner positive regulation (VCR)	Perceived partner negative regulation (VCR)	Duchenne smile (AC)	Overall cooperation in IPD
Valence (AC)	–									
Partner valence (AC)	.386**	–								
Valence (VCR)	.175	.116	–							
Perceived partner valence (VCR)	−.092	.279*	.461**	–						
Positive regulation (VCR)	.209	.209	.272*	.153	–					
Negative regulation (VCR)	.049	.044	.083	.064	.139	–				
Perceived partner positive regulation (VCR)	.222*	.325**	.197	.325**	.592**	.063	–			
Perceived partner negative regulation (VCR)	−.029	−.129	.059	−.061	.172	.673**	−.092	–		
Duchenne smile (AC)	.904**	.418**	.203	−.055	.293**	.089	.199	.013	–	
Overall cooperation in IPD	−.147	−.130	.286**	.386**	.145	.053	−.117	.185	−.109	–

*AC* auto-coded, *VCR* video-cued recall

*Correlation is significant at *p* < .05; **correlation is significant at *p* < .01

^+^Only participants with complete data for all correlations were included in the analysis

The only aggregated variables that correlated reliably with overall cooperation were VCR valence and perceived partner valence. This probably reflects the fact that actors and their partners responded positively to cooperation, although it is interesting that auto-coded valence and Duchenne smiling were (non-significantly) negatively correlated with cooperation levels, suggesting that the timing rather than frequency of smiling may be important for perceptions of valence. Similarly, auto-coded (AC) valence showed only small and non-significant positive correlations with VCR valence and positive regulation.

There was a significant positive correlation between actor and partner AC valence, suggesting that overall displayed facial positivity tended to correspond across participants in each dyad, probably due to interpersonal transfer of emotion (Parkinson, 2011), reciprocation of affiliative signals, or shared responses to mutual outcomes. Actor AC valence was also significantly associated with perceived partner positive regulation, suggesting that actors expressing more facial positivity were more likely to see their partner as upregulating their positive expressions. Similarly, partner AC valence was positively related with actor’s perceptions of partner positive regulation, suggesting that actors perceived high facial positivity as indicating upregulated positive expressions.

Finally, AC Duchenne smiles also showed a high positive correlation with AC valence. This mainly reflects the fact that AFFDEX’s computation of valence includes the action units associated with Duchenne smiles.

### Over-Time Dyadic Analyses

We ran a series of preregistered analyses assessing over-time effects of auto-coded facial signals and VCR ratings on decisions during the IPD games (see Table 5 for significant results, and Supplementary Materials for all results). The first analysis assessed lagged effects of actors’ and partners’ decisions to cooperate or defect on the actor’s decision in the subsequent game (hypothesis 1). We found a significant partner effect (*OR* = 2.601, *p* < .001, 95% CI [1.627, 4.157]), showing that participants were more than 2.5 times more likely to defect if their partner had defected on the previous trial. This partner effect remained significant in all subsequent analyses. This finding provides support for hypothesis 1 but only with respect to the effect of prior partner decisions and not prior actor decisions.

**Table 5:** Significant results from over-time (longitudinal) APIM, predicting participant’s decision (cooperate = 0, defect = 1)

Hypothesis	Lagged predictors	Odds ratios	*SE*	95% CI	*p*
1	Actor decision	1.371	.247	0.845–2.226	.201
	Partner decision	2.601	.239	1.627–4.157	**<.001**
3	Actor decision	1.600	.261	0.959–2.670	.072
	Partner decision	2.474	.254	1.505–4.068	**<.001**
	Positive regulation actor	1.006	.008	0.990–1.023	.446
	Positive regulation partner	.982	.008	0.966–.998	**.032**
	Negative regulation actor	1.001	.010	0.983–1.021	.876
	Negative regulation partner	1.003	.010	0.984–1.023	.737
4	Actor decision	1.525	.265	0.906–2.565	.112
	Partner decision	2.410	.255	1.461–3.976	**.001**
	Perceived positive regulation actor	.973	.009	0.955–.991	**.003**
	Perceived positive regulation partner	1.011	.009	0.993–1.029	.235
	Perceived negative regulation actor	.988	.010	0.968–1.008	.226
	Perceived negative regulation partner	1.011	.010	0.991–1.031	.288

Statistically significant (*α* = .05) *p*-values are marked in bold

Analyses 2–4 tested hypothesis 2 by assessing whether VCR and auto-coded valence ratings predicted subsequent actor cooperation controlling for prior actor and partner cooperation decisions. Analysis 2 included actor and partner VCR valence as predictors alongside both participants’ prior cooperation. Analysis 3 instead included actor and partner VCR perceived valence alongside prior cooperation. Analysis 4 included actor and partner AC valence alongside prior cooperation. None of the additional predictors reflecting valence or perceived valence were significant predictors in any of these models, so hypothesis 2 was not supported.

The fifth analysis tested hypothesis 3 by adding lagged VCR positive and negative regulation ratings made by actor and partner to lagged actor and partner cooperation as predictors of subsequent actor cooperation. Partner’s VCR positive regulation had a significant effect (*OR* = .982, *p* = .032, 95% CI [.966; .998]), showing that participants were significantly more likely to cooperate (less likely to defect) if their partner had rated their own positive regulation as higher in the previous round. Apart from the additional effect of partner’s decision, none of the other predictors had significant effects in this analysis. These findings are consistent with hypothesis 3 but only for VCR positive regulation and not for VCR negative regulation.

The sixth analysis tested hypothesis 4 by adding lagged actors’ and partners’ VCR perceptions of their partner’s positive and negative regulation to prior cooperation decisions as predictors of subsequent actor cooperation. The actor’s perceptions of their partner’s positive regulation had a significant effect (*OR* = .973, *p* = .003, 95% CI [.955; .991]), showing that participants were more likely to cooperate (less likely to defect) if they had rated their partner’s positive regulation as higher in the previous round. This finding conflicts with hypothesis 4’s prediction that perceived positive regulation would make subsequent cooperation less rather than more likely. Perceived negative regulation had no significant effect.

Hypothesis 5 was assessed in two further analyses. In the first of these, we added auto-coded Duchenne smiles to prior cooperation decisions as predictors of subsequent actor cooperation. In the second, we added our three auto-coded factor scores (reward smiles, mouth-based appeasement, and disapproval) alongside prior cooperation. None of these additional auto-coded predictors showed significant effects meaning that there was no support for hypothesis 5.

## Discussion

This study assessed over-time effects of auto-coded facial signals and VCR ratings of valence and facial regulation on cooperation during IPD games where participants interacted via video in real-time. Participants’ defection was positively predicted by their partner’s defection in the previous round, supporting hypothesis 1 and suggesting that players often adopted a tit-for-tat strategy (Rapoport & Chammah, 1965). Controlling for prior defection left little variance to explain, but we still found significant time-lagged effects of partner’s self-reported positive facial regulation and actor’s perception of this regulation, showing that cooperation was higher after partners strategically increased positive signalling (supporting hypothesis 3), or were perceived as so doing (going against hypothesis 4). Given that these predictors covered the period following the outcome of the previous trial not the seconds immediately preceding predicted decisions, these are impressive findings.

Actor-rated and partner-perceived positive regulation were not closely intercorrelated over time (see Hoegen et al., 2019). Thus, the lagged effect of perceived partner positive regulation did not depend on partner’s self-reported regulation. Our finding that perceived partner regulation encouraged subsequent actor cooperation goes against the prediction that participants would discount signals perceived as regulated because of their apparent manipulative intent (e.g., Côté et al., 2013; Shore & Parkinson, 2018). This discrepancy may reflect the fact that the present effects concerned regulation of positive rather than negative signals. Perceivers may have attributed regulated smiles to partner’s affiliative or apologetic intentions rather than any desire to deceive. Because our regulation measures ran from suppress to exaggerate, the effects may have depended on reduction of positive suppression rather than exaggeration, meaning that participants who relaxed (or were perceived as relaxing) down-regulatory attempts solicited greater cooperation. Disentangling these effects is difficult because separate measures of suppression and exaggeration are likely to intercorrelate.

Our study provided no support for hypotheses 2 and 5. These non-significant findings probably reflect the fact that partner’s prior defection already explained so much variance in subsequent actor cooperation. It is also possible that participants’ cooperative dispositions set the relational tone prior to gameplay, leaving little scope for further influence. Our dyads mostly engaged in mutual cooperation meaning that other influences needed to be particularly powerful to disrupt the interpersonal equilibrium. Future studies should use different payoff matrices to balance outcome probabilities more equally.

Between-participants correlations revealed that cooperation levels were not positively associated with AC partner valence or Duchenne smiling, unlike in previous research (e.g., Centorrino et al., 2015). However, de Melo et al. (2014) showed that equivalent smiling levels may encourage different levels of cooperation depending on the context for smile delivery. Similarly, we believe that it was when and why participants smiled not how much they smiled that made a difference to their partners’ cooperation. However, reported correlations may reflect responses to partner cooperation as well as its antecedents. Our over-time analyses provide more conclusive evidence about effect direction but revealed no evidence that facial activity predicted partners’ subsequent cooperation, consistent with the view that their interpersonal effects may vary over an extended interaction.

### Limitations and Future Directions

A central aim of this research was to develop methodology for investigating nonverbal communication and regulation during real-time interpersonal interactions. Our study addressed limitations of previous laboratory-based investigations of simulated, posed, non-interactive and/or static facial signals selected or manipulated to be pure expressions of “basic emotions.” Here, participants’ faces moved freely in real-time and any regulation could be attuned to dynamic feedback provided by the other person’s face.

Having captured interactive facial movements in the laboratory, the challenge was to measure them. Auto-coding already provides reasonably reliable data about individual AUs (see Method) with potential for further improvement especially when dealing with naturalistic behavior sequences (e.g., Gupta et al., 2022). Patterns of correlation between individual AUs may also be identified using principal component analysis (e.g., Robertson et al., 2023), but quantifying the possible dynamic transitions between identified facial configurations is more challenging. Future research should use dynamic facial movements as predictors of cooperation using time-series approaches.

Our VCR procedure was intended to approximate real-time perceptions of actor’s and partner’s regulation without disrupting gameplay as it happened. However, retrospective ratings may not fully capture ongoing experiences. Conceivably, participants’ recollection of the subsequent trial’s outcome may have biased their VCR ratings of positive regulation, undermining the apparent causal implications of their lagged effects. However, we think this unlikely for three reasons. First, our impression was that participants could not recall the exact sequence of outcomes. Second, participants were presented with video-clips of the periods immediately following the previous outcome and their main focus was on responses to that outcome rather than what would happen next. Third, even if participants recalled and were focused on the subsequent outcome during VCR, it seems unlikely that this would bias their ratings in accordance with our findings. Intuitive ideas about facial activity and cooperation would not seem to predict the reported effect of positive regulation. Substantiating our causal effect would require a real-time measure of regulation during the task, possibly based on dynamic patterns of actor-partner interdependency (e.g., Gratch, 2023). However, it is unclear whether such patterns would remain consistent across dyads or contexts. This also means that external observers would be unlikely to be sensitive to what goes on inside any particular dialogue.

To isolate effects of facial signals, we restricted communication to video displaying only participants’ heads and shoulders. However, facial movements outside the laboratory usually accompany speech and other gestural and postural cues (Parkinson, 2008). Constraining communicative possibilities may have meant that our participants used facial cues to fill in for absent channels. A fuller understanding of facial influence requires investigating its interactions with other communication modes. Finally, as cooperation and facial expressions differ across age and gender (e.g., McDuff et al., 2017; Vugt et al., 2007), future research should explore the effects of those variables.

## Conclusions

Our results suggest that regulating positive facial signals can encourage partners to cooperate and that those partners’ perception of regulation does not counteract this effect. These findings not only advance understanding of interpersonal influence but also illustrate how real-time dyadic measurement can clarify processes of emotion regulation during meaningful social interactions. Our research balanced experimental control with ecological validity to track processes that operate outside as well as inside the laboratory. Such an approach does not negate the importance of either more controlled experimental research or careful observation of more naturalistic facial interaction in the wild. Further clarification of how faces are regulated to optimise interpersonal influence requires a combination of methods and measures.

### Supplementary Information

ESM 1 (DOCX 89 kb)

## Footnotes

1 We did not systematically balance age or gender as our planned sample size was too small to provide sufficient power for the relevant comparisons.

2 We reran all analyses on the larger dataset (*N* = 98 from 48 dyads) that included participants who failed to meet this criterion (excluding only those trials on which iMotions data were missing). All of the significant effects reported below remained significant (with lower p values) in these analyses, suggesting that the findings are robust (see Supplementary Information for further details).

3 We also created “decision” clips and participants provided ratings of these during the VCR procedure too. Data from these decision clips were not analysed for the purposes of the present paper because it was difficult to determine which facial behaviors came before the decision to cooperate or defect and which reflected their subsequent responses to that decision.

## Additional Information

### Data availability

The data analysed in this paper, analysis code, and output can be accessed at https://osf.io/j59ne/files/osfstorage#.

### Code availability

Analysis code is available at https://osf.io/j59ne/files/osfstorage#.
