# Supplementary material for: Facial Regulation During Dyadic Interaction: Interpersonal Effects on Cooperation
Source: Affect Sci. 2023 Aug 23;4(3):506–16. doi: 10.1007/s42761-023-00208-y (PMC10514003; doi:10.1007/s42761-023-00208-y)
Supplement: Supplementary file 1 — (DOCX 89 kb) [file 42761_2023_208_MOESM1_ESM.docx]

Facial Regulation During Dyadic Interaction:
Interpersonal Effects on Cooperation and Trust – Supplementary Materials

Danielle Shore^1^, Olly Robertson^12^, Ginette Lafit^3, 4^, and Brian Parkinson^1^

^1^ Department of Experimental Psychology, University of Oxford, UK

^2^ Department of Psychiatry, University of Oxford, UK

^3^Research Group for Quantitative Psychology and Individual Differences, KU Leuven, Belgium
^4^Center for Contextual Psychiatry, Department of Neuroscience, KU Leuven, Belgium

# Author Note

This research was funded by the European Office of Aerospace Research and Development grant FA9550-18-1-0060 awarded to Brian Parkinson and Danielle Shore.

Correspondence concerning this article should be addressed to Brian Parkinson, Department of Experimental Psychology, University of Oxford, New Radcliffe House, Oxford OX6 2RR, UK. Email: brian.parkinson@psy.ox.ac.uk

# S1. Hypotheses

*Hypothesis 1*: Cooperation decisions will depend on the cooperation decision of the actor and partner in the previous round. An actor will be more likely to cooperate if the partner cooperated on the previous round.

*Hypothesis 2a*: Video-cued recall (VCR) ratings of expression valence (of both self and partner) will predict cooperation decision, over and above previous cooperation decision. Generally, more positive ratings will be associated with decisions to cooperate.

*Hypothesis 2b*: iMotions Valence scores will predict cooperation decision, over and above previous cooperation decision. Generally, higher scores (more positive/smile) will be associated with more cooperation.

*Hypothesis 2c*: Actor’s VCR ratings of partner’s expression valence will have greater ability to predict cooperation than iMotions partner Valence scores.

*Hypothesis 3a*: VCR ratings of regulation will influence cooperation decision, over and above previous cooperation decision.

*Hypothesis 3b*: VCR positive and negative regulation ratings will have independent effects on cooperation decisions.

*Hypothesis 4a*: The discrepancy in VCR ratings of expression valence (between partner and self) from the previous round will predict cooperation decision, over and above previous cooperation decision.

*Hypothesis 4b*: The discrepancy in VCR ratings of positive expression regulation (between partner and self) from the previous round will predict cooperation decision, over and above previous cooperation decision.

*Hypothesis 4c*: The discrepancy in VCR ratings of negative expression regulation (between partner and self) from the previous round will predict cooperation decision, over and above previous cooperation decision.

*Hypothesis 4d*: The discrepancy in VCR ratings of expression regulation (between partner and self) from the previous round will predict cooperation decision, over and above previous cooperation decision.

*Hypothesis 5*: Distinct expression scores from the previous round will predict cooperation decision, over and above previous cooperation decision.

*Hypothesis 6*: Likelihood score of expressed smile from iMotions will predict cooperation decision, over and above previous cooperation decision. Generally, higher scores (more smile) will be associated with more cooperation.

*Hypothesis 7*: Likelihood score of expressed smile from iMotions will predict cooperation decision, over and above previous cooperation decision, while controlling for previous positive regulation scores (VCR rating). Generally, higher scores (more smile) will be associated with more cooperation.

**Note.** Following initial analyses, we updated the preregistration to include follow-up analyses to better understand and clarify the results. However, we detected a problem with the sequencing of entries in our datafile. Rerunning analyses on the corrected datafile revealed a partly different pattern of effects, which meant that the preregistered follow-up analyses covered by Hypotheses 4a to 4d were no longer relevant. However, those hypotheses and the associated analyses are included in these Supplementary Materials for the sake of transparency.

# S2. Post-Task Questionnaire Measure

## Participant VCR Ratings of Perceived and Actual Expression Valence

Participants were presented with the 5-second video clip starting from the presentation of the round outcome (i.e., when the round outcome was revealed) and rated the extent to which they were expressing their positive and negative expressions separately. They also completed the same ratings for perceived opponent expression. These ratings were made on a round-by-round basis using a series of visual analogue scale anchored from -50 (‘Negative’) to 50 (‘Positive’). Participants completed ratings of perceived and actual expression valence for each round using the following items.

1. How positive/negative was the emotion that you were expressing?
2. How positive/negative was the emotion that your opponent was expressing?

Participants also completed the same ratings for perceived opponent regulation. These ratings were made on a round-by-round basis using a series of visual analogue scale anchored from -50 (‘Suppressed’) to 50 (‘Exaggerated’). Participants completed ratings of perceived and actual regulation for each round using the following items.

2a. To what extent were you regulating your facial expressions of positive emotions?

2b. To what extent were you regulating your facial expressions of negative emotions?

4a. To what extent was your opponent regulating their facial expressions of positive emotions?

4b. To what extent was your opponent regulating their facial expressions of negative emotions?

## Post-Recall Questions

Participants were presented with a series of questions regarding their overall experiences of gameplay and impressions of their partner after the video cued recall questions were completed.

Participants were asked to rate (“To what extent do you think your opponent is:“) the following items on a 7-point Likert scale anchored from ‘Not at all’ to ‘Extremely’:

- Friendly
- Happy
- Unkind
- Trustworthy
- Attractive
- Sad
- Dominant
- Disagreeable

Participants were also asked to rate the extent to which they agreed with the following statements (“Please rate the extent to which you agree with the following statements:”) on a 5-point Likert scale, anchored from ‘Not at all’ to ‘Extremely’:

- My opponent was faking how they felt
- My opponent was pretending or putting on an act
- My opponent genuinely expressed their emotions
- My opponent was sincere
- The emotions that my opponent expressed were not real
- My opponent expressed emotions they did not really feel inside
- In my opinion my opponent is reliable
- I think my opponent expressed the truth
- I think my opponent was honest
- I think my opponent acted in line with their expressions
- I think my opponent took advantage of me
- I do not think my opponent misled me
- I was faking how I felt
- I was pretending or putting on an act
- I genuinely expressed my emotions
- I was sincere
- The emotions that I expressed were not real
- I expressed emotions I did not really feel inside

Participants were asked to rate their answers to the following questions on a 7-point Likert scale, anchored from ‘Extremely Unfairly’ to ‘Extremely Fairly’:

- How fairly did **your opponent** behave?
- How fairly did **you** behave?

Participants were asked to rate their answers to the following questions on a 7-point Likert scale, anchored from ‘Not at all’ to ‘Extremely’:

- How cooperative was **your opponent**?
- How cooperative were **you**?
- To what extent did **you** trust **your opponent**?
- To what extent did **you** trust **your opponent**?

# S3. Analyses

For additional details, please see the preregistered analysis plan: <https://osf.io/dzrc3>

In the study, repeated measurements (i.e., participants play 10 rounds of a computer-mediated iterated prisoner’s dilemma game “split-steal”) are nested within indistinguishable partners. To consider this structure, we used two-levels mixed-effects models. All multilevel models were estimated using R packages lme4 (Bates et al., 2015).

## Model 1 (Hypothesis 1)

To test hypothesis 1, we estimated a Longitudinal Actor-Partner Interdependence Model (L-APIM). We fitted a mixed-effects logistic regression, where the outcome was a binary variable indicating the participant’s cooperation level (Cooperation_Level) at the t-th round (0=Cooperate, 1=Defect), and the predictors were their own decision in the previous round (actor effect), and their partner’s decision in the previous round (partner effect). We assumed that partners are indistinguishable, thus the sizes of both actor and partner effects do not differ across partners. To account for differences across dyads, we introduced a random effect for the intercept.

The syntax of Model 1 is given by:

Model1 = glmer(Cooperation_Level ~ Actor_Cooperation_Level_Lag +

Partner_Cooperation_Level_Lag + (1|Dyad_ID), data, family = binomial)

where “Cooperation_Level” is the binary outcome indicating the participant’s decision at the t-th round, Actor_Cooperation_Level_Lag” indicates the participant’s own decision in the previous round, and “Partner_Cooperation_Level_Lag” indicates their partner’s decision in the previous round. The variable "Dyad_ID" indicates the dyad's identification number.

## Models 2a.1 and 2a.2 (Hypothesis 2a)

To test hypothesis 2a.1, we added two further predictors to model 1: the participant’s own VCR ratings of expression valence i.e., "How positive/negative was the emotion that you were expressing?” (Actor_Rating_Valence), and the partner’s VCR ratings of their own expression valence (Partner_Rating_Valence).

The syntax of this model is:

Model2a.1 = glmer(Cooperation_Level ~ Actor_Cooperation_Level_Lag + Partner_Cooperation_Level_Lag + Actor_Rating_Valence + Partner_Rating_Valence + (1|Dyad_ID), data, family = binomial)

To test hypothesis 2a.2, we added two further predictors to model 1: the participant’s VCR ratings of the expression valence of their partner i.e., “How positive/negative was the emotion that your opponent was expressing?” (Actor_Rating_Valence_Partner), the partner video ratings of their opponent’s (i.e., actor) expression valence (Partner_Rating_Valence_Partner).

The syntax of this model is:

Model2a.2 = glmer(Cooperation_Level ~ Actor_Cooperation_Level_Lag + Partner_Cooperation_Level_Lag + Actor_Rating_Valence_Partner + Partner_Rating_Valence_Partner+ (1|Dyad_ID), data, family = binomial)

## Model 2b (Hypothesis 2b)

To test hypothesis 2b, we added the participant’s own iMotions Valence score (Actor_iMotions_Valence), and the partner’s iMotions Valence score (Partner_iMotions_Valence) to model 1.

The model syntax is:

Model2b = glmer(Cooperation_Level ~ Actor_Cooperation_Level_Lag + Partner_Cooperation_Level_Lag + Actor_iMotions_Valence + Partner_ iMotions _Valence + (1|Dyad_ID), data, family = binomial)

## Model 2c (Hypothesis 2c)

We tested hypothesis 2c by extending model 1 to include the participant’s VCR rating of their partner’s expression valence (Actor_Rating_Valence_Partner), their own iMotions Valence score (Actor_iMotions_Valence), their partner’s VCR rating of the actor’s expression valence (Partner_Rating_Valence_Partner), and the partner’s iMotions Valence score (Partner_iMotions_Valence).

Model 2c can be represented as:

Model2c = glmer(Cooperation_Level ~ Actor_Cooperation_Level_Lag + Partner_Cooperation_Level_Lag + Actor_Rating_Valence_Partner + Partner_iMotions_Valence + Partner_ Rating_Valence_Partner + Actor_iMotions_Valence + (1|Dyad_ID), data, family = binomial)

To assess predictive power of each of the predictors, we compared the odd ratios associated with the actor’s VCR ratings of their partner’s expression valence (Actor_Rating_Valence_Partner) with those of the iMotions partner Valence scores (Partner_ iMotions _Valence).

## Models 3a.1 and 3a.2 (Hypothesis 3a)

To test hypothesis 3a, we fitted two separate models. The first model, extended model 1 by including the participant's VCR ratings of their own regulation (i.e., To what extent were you regulating your facial expression?) and their partner’s VCR ratings of their own regulation (denoting the actor and partner effects as Actor_Regulation and Partner_Regulation, respectively).

The first model is represented as:

Model3a.1 = glmer(Cooperation_Level ~ Actor_Cooperation_Level_Lag + Partner_Cooperation_Level_Lag + Actor_Regulation + Partner_Regulation + (1|Dyad_ID), data, family = binomial)

For the second model, model 1 was extended by including the participant's VCR rating of their partner’s regulation (To what extent was your opponent regulating their facial expressions?) which is denoted by Actor_Regulation_Partner, and their partner’s VCR rating of the actor’s regulation (Partner_Regulation_Partner).

The model is given by:

Model3a.2 = glmer(Cooperation_Level ~ Actor_Cooperation_Level_Lag + Partner_Cooperation_Level_Lag + Actor_Regulation_Partner + Partner_Regulation_Partner + (1|Dyad_ID), data, family = binomial)

## Models 3b.1 and 3b.2 (Hypothesis 3b)

Hypothesis 3b was tested by estimating two separate models. The first model extended model 1 by including the participant's VCR ratings of their own positive and negative regulation (i.e., To what extent were you regulating your facial expressions of positive/negative emotions?) and their partner’s VCR ratings of their own positive and negative regulation (denoting the actor and partner effects as Actor_Positive_Regulation, Actor_Negative_Regulation, and Partner_Positive_Regulation, Partner_Negative_Regulation).

The first model is represented as:

Model3b.1 = glmer(Cooperation_Level ~ Actor_Cooperation_Level_Lag + Partner_Cooperation_Level_Lag + Actor_Positive_Regulation + Actor_Negative_Regulation + Partner_Positive_Regulation + Partner_Negative_Regulation + (1|Dyad_ID), data, family = binomial)

To assess predictive power of each of the predictors, we compared the odd ratios associated with actor and partner effects of positive and negative regulation. Subsequently, we estimated a model including the participant's VCR ratings of their partner's positive and negative regulation (i.e., To what extent was your opponent regulating their facial expressions of positive emotions?) and their partner’s self-rated positive and negative regulation (denoting the actor and partner effects as Actor_Positive_Regulation_Partner, Actor_Negative_Regulation_Partner, and Partner_Positive_Regulation_Partner, Partner_Negative_Regulation_Partner).

The model is given by:

Model3b.2 = glmer(Cooperation_Level ~ Actor_Cooperation_Level_Lag + Partner_Cooperation_Level_Lag + Actor_Positive_Regulation_Partner + Actor_Negative_Regulation_Partner + Partner_Positive_Regulation_Partner + Partner_Negative_Regulation_Partner + (1|Dyad_ID), data, family = binomial)

To assess predictive power of each of the predictors we compared the odd ratios associated with actor and partner effects of positive and negative regulation.

## Models 4a and 4b (Hypotheses 4a and 4b)

To test hypotheses 4a to 4b, we assumed a two-level structure where repeated measurements are nested within participants. To test hypothesis 4a, we estimated a mixed-effects logistic regression, where the outcome is a binary variable indicating the participant cooperation level (Cooperation_Level) at the t-th round (0=Cooperate, 1=Defect), and the predictors are their own decision in the previous round (actor effect), their partner’s decision in the previous round (partner effect), and the participant’s discrepancy in video ratings of expression valence (between partner and self) from the previous round (discrepancyValence_Lag). To account for differences across persons, we introduced a random effect for the intercept.

The syntax of this model is:

Model4a = glmer(Cooperation_Level ~ Actor_Cooperation_Level_Lag + Partner_Cooperation_Level_Lag + discrepancyValence_Lag + (1|Person_ID), data, family = binomial)

The variable "Person_ID" indicates the participant’s identification number. To test hypothesis 4b, we modified model 4a by including the participant’s discrepancy in video ratings of positive expression regulation (between partner and self) from the previous round (discrepancyPositiveReg_Lag).

The syntax of this model is:

Model4b = glmer(Cooperation_Level ~ Actor_Cooperation_Level_Lag + Partner_Cooperation_Level_Lag + discrepancyPositiveReg_Lag + (1|Person_ID), data, family = binomial)

## Model 4c (Hypothesis 4c)

Similar to the previous models, we tested hypothesis 4c by including the participant’s discrepancy in video ratings of negative expression regulation (between partner and self) from the previous round (discrepancyNegativeReg_Lag):

Model4c = glmer(Cooperation_Level ~ Actor_Cooperation_Level_Lag + Partner_Cooperation_Level_Lag + discrepancyNegativeReg_Lag + (1|Person_ID), data, family = binomial)

## Model 4d (Hypothesis 4d)

We tested hypothesis 4d by fitting a logistic mixed-effects model including the participant’s discrepancy in video ratings of expression regulation (between partner and self) from the previous round (discrepancyRegulation_Lag), controlling for the participant’s and their partner’s cooperation level at the previous round:

Model4d = glmer(Cooperation_Level ~ Actor_Cooperation_Level_Lag + Partner_Cooperation_Level_Lag + discrepancyRegulation_Lag + (1|Person_ID), data, family = binomial)

## Model 5 (Hypothesis 5)

To test hypothesis 5, we estimated a Longitudinal Actor-Partner Interdependence Model (L-APIM). We fitted a mixed-effects logistic regression, where the outcome was a binary variable indicating the participant cooperation level (Cooperation_Level) at the t-th round (0=Cooperate, 1=Defect), and the predictors were their own decision in the previous round (actor effect), their partner’s decision in the previous round (partner effect), the participant’s distinct expression scores at the previous round (Reward_Smile_Lag, Mouth_based_Appeasement_Lag, and Disapproval_Lag^[[1]](#footnote-1)^), and the partner’s distinct expression scores at the previous round (Reward_Smile_Lag, Mouth_based_Appeasement_Lag, and Disapproval_Lag). We assumed that partners are indistinguishable, thus the sizes of both actor and partner effects do not differ across partners. To account for differences across dyads, we introduced a random effect for the intercept.

The syntax of this model is:

Model5 = glmer(Cooperation_Level ~ Actor_Cooperation_Level_Lag + Partner_Cooperation_Level_Lag + Actor_Reward_Smile_Lag + Actor_Mouth_based_Appeasement_Lag + Actor_Disapproval_Valence_Lag + Partner_Reward_Smile_Lag + Partner_Mouth_based_Appeasement_Lag + Partner_Disapproval_Valence_Lag + (1|Dyad_ID), data, family = binomial)

## Model 6 (Hypothesis 6)

To test hypothesis 6, we estimated a Longitudinal Actor-Partner Interdependence Model (L-APIM). We fitted a mixed-effects logistic regression, where the outcome was a binary variable indicating the participant’s cooperation level (Cooperation_Level) at the t-th round (0=Cooperate, 1=Defect), and the predictors were their own decision in the previous round (actor effect), their partner’s decision in the previous round (partner effect), the actor’s iMotions Duchenne Smile score, and the partner’s iMotions Duchenne Smile score. We assumed that partners are indistinguishable, thus the sizes of both actor and partner effects do not differ across partners. To account for differences across dyads, we introduced a random effect for the intercept.

The syntax of this model is:

Model6 = glmer(Cooperation_Level ~ Actor_Cooperation_Level_Lag + Partner_Cooperation_Level_Lag + Actor_Smile_Lag + Partner_Smile_Lag +

(1|Dyad_ID), data, family = binomial)

## Model 7 (Hypothesis 7)

To test hypothesis 7, we extended Model 6 by including the participant's VCR rating of positive regulation (i.e., To what extent were you regulating your facial expressions of positive emotions?) and their partner’s VCR rating of their own positive regulation (denoting the actor and partner effects as Actor_Positive_Regulation and Partner_Positive_Regulation).

The syntax of this model is:

Model6 = glmer(Cooperation_Level ~ Actor_Cooperation_Level_Lag + Partner_Cooperation_Level_Lag + Actor_Smile_Lag + Partner_Smile_Lag + Actor_Positive_Regulation + Partner_Positive_Regulation +

(1|Dyad_ID), data, family = binomial)

# S4. Analysis

For code and html Rmarkdown files, please see the preregistered project: [osf.io/j59ne](https://osf.io/j59ne)

## **Descriptive statistics**

| *Table S1.* Descriptive statistics (N_Person_ = 90, N_Dyad_ = 45). | |  |  |  |  |  |
| --- | --- | --- | --- | --- | --- | --- |
| Variable | | Mean | SD | Median | Min | Max |
| Age | | 26.64 | 7.57 | 24 | 18 | 59 |
| Gender (% female) | | 67.8 |  |  |  |  |
| Gender (% male) | | 32.2 |  |  |  |  |
| Ethnicity (%) | |  |  |  |  |  |
|  | Caucasian | 76.7% |  |  |  |  |
|  | Mixed | 5.6% |  |  |  |  |
|  | Asian | 8.9% |  |  |  |  |
|  | Black | 3.3% |  |  |  |  |
|  | Other | 5.6% |  |  |  |  |
| Decision (% defect) | | 25.44 |  |  |  |  |
| Mutual cooperation (%) | | 60.67 |  |  |  |  |
| Mutual defection (%) | | 11.56 |  |  |  |  |
| Valence (AC) | | 15.64 | 30.76 | 1.10 | -93.77 | 99.63 |
| Valence (VCR; q1) | | 8.75 | 17.12 | 6 | -50 | 50 |
| Positive regulation (VCR; Q2a) | | -3.53 | 15.49 | 0 | -50 | 50 |
| Negative regulation (VCR; Q2b) | | -3.61 | 12.05 | 0 | -50 | 50 |
| Perceived partner valence (VCR; Q3) | | 8.78 | 16.20 | 6 | -46 | 50 |
| Perceived partner positive regulation (VCR; q4a) | | -2.16 | 15.27 | 0 | -50 | 44 |
| Perceived partner positive regulation (VCR; q4b) | | -4.32 | 11.66 | 0 | -50 | 50 |
| Actor Regulation (Sum of q2a and q2bReverse) | | 0.08 | 20.05 | 0 | -69 | 91 |
| Partner Regulation (Sum of q4a and q4bReverse) | | 2.16 | 20.10 | 0 | -79 | 82 |
| Discrepancy Valence | | 0.01 | 20.72 | 0 | -81 | 84 |
| Discrepancy Positive Regulation | | 1.37 | 20.32 | 0 | -56 | 61 |
| Discrepancy Negative Regulation | | -0.71 | 15.35 | 0 | -59 | 64 |
| Discrepancy Regulation | | 2.08 | 26.64 | 0 | -87 | 99 |
| Duchenne Smile (AC) | | 28.12 | 32.33 | 12.64 | 0 | 100 |
| Reward smile (AC) | | 21.83 | 24.79 | 11.51 | 0 | 99.95 |
| Mouth-based appeasement (AC) | | 8.28 | 11.43 | 3.55 | 0 | 76.38 |
| Disapproval (AC) | | 1.54 | 4.48 | 0.04 | 0 | 33.66 |
| *Note.* AC = Auto-Coded; VCR = Video Cued Recall; Q = Question, from post task questionnaire measures (see *S2*); Reward smile, Mouth-based appeasement, and disapproval expressions dervied from Robertson et al. (*in prep*), see <https://osf.io/v76ha/>. | | | | | | |

##

## **Results of the over-time (longitudinal) APIM**

*Table S2*. Table 3. Results of the over-time (longitudinal) APIM, predicting participant’s decision to defect from their previous round decision to cooperate or defect. Results also include the lagged values of video ratings of expression valence, iMotions valence scores, video ratings, and likelihood score of expressed smile from facial muscle movement.

| Model | Lagged predictors | Odd ratios | SE | 95% CI | *p* |
| --- | --- | --- | --- | --- | --- |
| 1 | Actor decision | 1.371 | 0.247 | 0.845 – 2.226 | 0.201 |
|  | Partner decision | 2.601 | 0.239 | 1.627 – 4.157 | **<0.001** |
| 2.a1 | Actor decision | 1.257 | 0.259 | 0.756 – 2.089 | 0.378 |
|  | Partner decision | 2.537 | 0.250 | 1.554 – 4.142 | **<0.001** |
|  | Actor Valence (VCR; Q1) | 0.997 | 0.007 | 0.983 – 1.011 | 0.659 |
|  | Partner Valence (VCR; Q1) | 0.992 | 0.007 | 0.978 – 1.006 | 0.253 |
| 2.a2 | Actor decision | 1.366 | 0.254 | 0.830 – 2.248 | 0.220 |
|  | Partner decision | 2.423 | 0.246 | 1.496 – 3.925 | **<0.001** |
|  | Actor perceived partner valence (VCR; Q3) | 0.996 | 0.008 | 0.981 – 1.011 | 0.598 |
|  | Partner perceived partner valence (VCR; Q3) | 0.988 | 0.008 | 0.973 – 1.002 | 0.103 |
| 2.b | Actor decision | 1.342 | 0.271 | 0.788 – 2.280 | 0.279 |
|  | Partner decision | 2.569 | 0.264 | 1.531 – 4.310 | **<0.001** |
|  | Actor Valence (AC) | 0.996 | 0.004 | 0.987 – 1.004 | 0.330 |
|  | Partner Valence (AC) | 0.994 | 0.004 | 0.986 – 1.003 | 0.178 |
| 2.c | Actor decision | 1.416 | 0.288 | 0.806 – 2.489 | 0.226 |
|  | Partner decision | 2.684 | 0.280 | 1.549 – 4.650 | **<0.001** |
|  | Actor perceived partner valence (VCR; Q3) | 0.996 | 0.009 | 0.979 – 1.012 | 0.602 |
|  | Partner perceived partner valence (VCR; Q3) | 0.990 | 0.009 | 0.974 – 1.007 | 0.256 |
|  | Actor Valence (AC) | 0.996 | 0.005 | 0.987 – 1.006 | 0.443 |
|  | Partner Valence (AC) | 0.995 | 0.005 | 0.986 – 1.004 | 0.299 |
| 3.a1 | Actor decision | 1.613 | 0.261 | 0.968 – 2.688 | 0.067 |
|  | Partner decision | 2.440 | 0.253 | 1.486 – 4.004 | **<0.001** |
|  | Actor regulation (VCR; Sum of q2a and q2bReverse) | 1.003 | 0.006 | 0.992 – 1.015 | 0.573 |
|  | Partner regulation (VCR; Sum of q2a and q2bReverse) | 0.988 | 0.006 | 0.977 – 1.000 | 0.050 |

*Table S2 (continue).* Results of the over-time (longitudinal) APIM, predicting participant’s decision to defect from their previous round decision to cooperate or defect. Results also include the lagged values of video ratings of expression valence, iMotions valence scores, and video ratings.

| Model | Lagged predictors | Odd ratios | SE | 95% CI | *p* |
| --- | --- | --- | --- | --- | --- |
| 3.a2 | Actor decision | 1.519 | 0.261 | 0.911 – 2.531 | 0.109 |
|  | Partner decision | 2.426 | 0.253 | 1.476 – 3.987 | **<0.001** |
|  | Actor’s perceived partner regulation (VCR; Sum of q4a and q4bReverse) | 0.991 | 0.007 | 0.978 – 1.004 | 0.158 |
|  | Partner’s perceived partner regulation (VCR; Sum of q4a and q4bReverse) | 1.000 | 0.006 | 0.988 – 1.013 | 0.940 |
| 3.b1 | Actor decision | 1.600 | 0.261 | 0.959 – 2.670 | 0.072 |
|  | Partner decision | 2.474 | 0.254 | 1.505 – 4.068 | **<0.001** |
|  | Actor positive regulation (VCR; Q2a) | 1.006 | 0.008 | 0.990 – 1.023 | 0.446 |
|  | Partner positive regulation (VCR; Q2a) | 0.982 | 0.008 | 0.966 – 0.998 | **0.032** |
|  | Actor negative regulation (VCR; Q2b) | 1.001 | 0.010 | 0.983 – 1.021 | 0.876 |
|  | Partner negative regulation (VCR; Q2b) | 1.003 | 0.010 | 0.984 – 1.023 | 0.737 |
| 3.b2 | Actor decision | 1.525 | 0.265 | 0.906 – 2.565 | 0.112 |
|  | Partner decision | 2.410 | 0.255 | 1.461 – 3.976 | **0.001** |
|  | Actor’s perceived partner positive regulation (VCR; q4a) | 0.973 | 0.009 | 0.955 – 0.991 | **0.003** |
|  | Partner’s perceived partner positive regulation (VCR; q4a) | 1.011 | 0.009 | 0.993 – 1.029 | 0.235 |
|  | Actor’s perceived partner negative regulation (VCR; q4b) | 0.988 | 0.010 | 0.968 – 1.008 | 0.226 |
|  | Partner’s perceived partner negative regulation (VCR; q4b) | 1.011 | 0.010 | 0.991 – 1.031 | 0.288 |
| 5 | Actor decision | 1.296 | 0.278 | 0.752 – 2.235 | 0.351 |
|  | Partner decision | 2.578 | 0.271 | 1.515 – 4.386 | **<0.001** |
|  | Actor reward smile | 0.996 | 0.007 | 0.983 – 1.010 | 0.569 |
|  | Actor mouth-based appeasement | 0.992 | 0.012 | 0.969 – 1.015 | 0.486 |
|  | Actor disapproval | 1.015 | 0.040 | 0.938 – 1.097 | 0.715 |
|  | Partner reward smile | 0.991 | 0.007 | 0.978 – 1.005 | 0.195 |
|  | Partner mouth-based appeasement | 1.007 | 0.012 | 0.985 – 1.030 | 0.532 |
|  | Partner disapproval | 1.025 | 0.039 | 0.949 – 1.108 | 0.522 |

*Note*. Statistically significant (α = .05) p-values are marked in bold; AC = Auto-Coded; VCR = Video Cued Recall; Q = Question, from post task questionnaire measures (see S2); see S3 for model information; Reward smile, mouth-based appeasement, and disapproval expressions dervied from Robertson et al. (*in prep*).

## **Results of logistic mixed-effect models**

*Table S3*. Results of logistic mixed-effect models, predicting participant’s decision to defect from their previous round decision to cooperate or defect and the discrepancy (between partner and self) ratings.

| Model | Lagged predictors | Odd ratios | SE | 95% CI | *p* |
| --- | --- | --- | --- | --- | --- |
| 4.a | Actor decision | 2.701 | 0.322 | 1.437 – 5.074 | **0.002** |
|  | Partner decision | 5.873 | 0.241 | 3.659 – 9.426 | **<0.001** |
|  | Discrepancy Actor Valence | 0.995 | 0.006 | 0.984 – 1.006 | 0.392 |
| 4.b | Actor decision | 2.719 | 0.322 | 1.447 – 5.109 | **0.002** |
|  | Partner decision | 5.821 | 0.242 | 3.619 – 9.361 | **<0.001** |
|  | Discrepancy Actor positive regulation | 1.001 | 0.007 | 0.988 – 1.014 | 0.906 |
| 4.c | Actor decision | 2.711 | 0.316 | 1.458 – 5.039 | **0.002** |
|  | Partner decision | 5.871 | 0.240 | 3.666 – 9.403 | **<0.001** |
|  | Discrepancy Actor negative regulation | 0.990 | 0.007 | 0.976 – 1.005 | 0.182 |
| 4.d | Actor decision | 2.759 | 0.319 | 1.476 – 5.158 | **0.001** |
|  | Partner decision | 5.930 | 0.241 | 3.700 – 9.505 | **<0.001** |
|  | Discrepancy Actor regulation | 1.004 | 0.005 | 0.995 – 1.014 | 0.354 |

*Note*. Statistically significant (α = .05) p-values are marked in bold; see S3 for model information

*Table S4*. Results of the over-time (longitudinal) APIM, predicting participant’s decision to defect from their previous round decision to cooperate or defect. Results also include the lagged values of video ratings of expression valence, and likelihood score of expressed smile from facial muscle movement.

| Model | Lagged predictors | Odd ratios | SE | 95% CI | *p* |
| --- | --- | --- | --- | --- | --- |
| 6 | Actor decision | 1.335 | 0.270 | 0.786 – 2.267 | 0.284 |
|  | Partner decision | 2.559 | 0.263 | 1.529 – 4.281 | **<0.001** |
|  | Actor Duchenne smile | 0.995 | 0.005 | 0.985 – 1.005 | 0.294 |
|  | Partner Duchenne smile | 0.994 | 0.005 | 0.984 – 1.004 | 0.208 |
| 7 | Actor decision | 1.553 | 0.286 | 0.887 – 2.718 | 0.124 |
|  | Partner decision | 2.273 | 0.275 | 1.325 – 3.901 | **0.003** |
|  | Actor Duchenne smile | 0.993 | 0.005 | 0.983 – 1.004 | 0.206 |
|  | Partner Duchenne smile | 0.996 | 0.005 | 0.985 – 1.006 | 0.402 |
|  | Actor positive regulation (VCR; Q2a) | 1.018 | 0.009 | 1.001 – 1.036 | **0.042** |
|  | Partner positive regulation (VCR; Q2a) | 0.982 | 0.009 | 0.965 – 1.000 | **0.046** |

*Note*. Statistically significant (α = .05) p-values are marked in bold; see S3 for model information.

**S5. Sensitivity analyses**

We conducted the analyses on the complete data set without excluding participants based on the proportion of missing iMotions data (i.e., for each participant and each round observations were set as missing if iMotions recognised a face is in the frame, but was unable to analyse the face for Action Units). The analyses included 98 participants and 49 dyads.

## **Descriptive statistics**

| *Table S5.* Descriptive statistics (N_Person_ = 98, N_Dyad_ = 49). | | | | | |
| --- | --- | --- | --- | --- | --- |
| Variable | Mean | SD | Median | Min | Max |
| Decision (% defect) | 24.08 |  |  |  |  |
| Mutual cooperation (%) | 62.45 |  |  |  |  |
| Mutual defection (%) | 10.61 |  |  |  |  |
| Valence (AC) | 18.28 | 32.85 | 2.74 | -93.74 | 99.62 |
| Valence (VCR; q1) | 8.66 | 16.88 | 6 | -50 | 50 |
| Positive regulation (VCR; Q2a) | -3.21 | 15.30 | 0 | -50 | 50 |
| Negative regulation (VCR; Q2b) | -3.55 | 11.78 | 0 | -50 | 50 |
| Perceived partner valence (VCR; Q3) | 8.82 | 16.24 | 6 | -46 | 50 |
| Perceived partner positive regulation (VCR; q4a) | -2.14 | 15.09 | 0 | -50 | 44 |
| Perceived partner positive regulation (VCR; q4b) | -4.50 | 11.67 | 0 | -50 | 50 |
| Actor Regulation (Sum of q2a and q2bReverse) | 0.34 | 19.84 | 0 | -69 | 91 |
| Partner Regulation (Sum of q4a and q4bReverse) | 2.36 | 20.08 | 0 | -79 | 82 |
| Discrepancy Valence | 0.13 | 20.92 | 0 | -81 | 84 |
| Discrepancy Positive Regulation | 1.16 | 20.05 | 0 | -56 | 62 |
| Discrepancy Negative Regulation | -0.93 | 15.19 | 0 | -59 | 64 |
| Discrepancy Regulation | 2.09 | 26.41 | 0 | -87 | 100 |
| Duchenne Smile (AC) | 29.22 | 32.51 | 14.97 | 0 | 100 |
| Reward smile (AC) | 22.92 | 25.37 | 13.34 | 0 | 99.95 |
| Mouth-based appeasement (AC) | 8.54 | 11.58 | 3.78 | 0 | 76.38 |
| Disapproval (AC) | 1.59 | 4.5 | 0.05 | 0 | 33.73 |
| *Note.* AC = Automated Coding; VCR = Video Cued Recall; Q = Question, from post task questionnaire measures (see *S2*); Reward smile, Mouth-based appeasement, and disapproval expressions dervied from Robertson et al., (*in prep*), see <https://osf.io/v76ha/>. | | | | | |

##

## **Results of the over-time (longitudinal) APIM**

*Table S6*. Results of the over-time (longitudinal) APIM, predicting participant’s decision to defect from their previous round decision to cooperate or defect. Results also include the lagged values of video ratings of expression valence, iMotions valence scores, video ratings, and likelihood score of expressed smile from facial muscle movement.

| Model | Lagged predictors | Odd ratios | SE | 95% CI | *p* |
| --- | --- | --- | --- | --- | --- |
| 1 | Actor decision | 1.286 | 0.241 | 0.802 – 2.064 | 0.296 |
|  | Partner decision | 2.389 | 0.233 | 1.513 – 3.772 | **<0.001** |
| 2.a1 | Actor decision | 1.202 | 0.255 | 0.730 – 1.979 | 0.471 |
|  | Partner decision | 2.429 | 0.246 | 1.501 – 3.931 | **<0.001** |
|  | Actor Valence (VCR; Q1) | 0.999 | 0.007 | 0.985 – 1.012 | 0.847 |
|  | Partner Valence (VCR; Q1) | 0.992 | 0.007 | 0.978 – 1.006 | 0.252 |
| 2.a2 | Actor decision | 1.308 | 0.25 | 0.801 – 2.135 | 0.283 |
|  | Partner decision | 2.280 | 0.242 | 1.419 – 3.663 | **0.001** |
|  | Actor perceived partner valence (VCR; Q3) | 0.997 | 0.007 | 0.983 – 1.011 | 0.683 |
|  | Partner perceived partner valence (VCR; Q3) | 0.989 | 0.007 | 0.975 – 1.003 | 0.114 |
| 2.b | Actor decision | 1.343 | 0.247 | 0.828 – 2.177 | 0.232 |
|  | Partner decision | 2.498 | 0.239 | 1.563 – 3.990 | **<0.001** |
|  | Actor Valence (AC) | 0.997 | 0.004 | 0.989 – 1.004 | 0.377 |
|  | Partner Valence (AC) | 0.999 | 0.004 | 0.991 – 1.006 | 0.742 |
| 2.c | Actor decision | 1.336 | 0.233 | 0.846 – 2.109 | 0.214 |
|  | Partner decision | 2.337 | 0.226 | 1.500 – 3.642 | **<0.001** |
|  | Actor perceived partner valence (VCR; Q3) | 0.997 | 0.007 | 0.983 – 1.012 | 0.718 |
|  | Partner perceived partner valence (VCR; Q3) | 0.989 | 0.007 | 0.975 – 1.004 | 0.142 |
|  | Actor Valence (AC) | 0.998 | 0.004 | 0.990 – 1.006 | 0.588 |
|  | Partner Valence (AC) | 1.000 | 0.004 | 0.992 – 1.008 | 0.932 |
| 3.a1 | Actor decision | 1.535 | 0.256 | 0.930 – 2.534 | 0.094 |
|  | Partner decision | 2.186 | 0.248 | 1.345 – 3.551 | **0.002** |
|  | Actor regulation (VCR; Sum of q2a and q2bReverse) | 1.007 | 0.006 | 0.995 – 1.018 | 0.251 |
|  | Partner regulation (VCR; Sum of q2a and q2bReverse) | 0.989 | 0.006 | 0.978 – 1.001 | 0.067 |

*Table S6 (continued).* Results of the over-time (longitudinal) APIM, predicting participant’s decision to defect from their previous round decision to cooperate or defect. Results also include the lagged values of video ratings of expression valence, iMotions valence scores, and video ratings.

| Model | Lagged predictors | Odd ratios | SE | 95% CI | *p* |
| --- | --- | --- | --- | --- | --- |
| 3.a2 | Actor decision | 1.506 | 0.001 | 1.503 – 1.509 | **<0.001** |
|  | Partner decision | 2.247 | 0.001 | 2.243 – 2.251 | **<0.001** |
|  | Actor’s perceived partner regulation (VCR; Sum of q4a and q4bReverse) | 0.988 | 0.001 | 0.986 – 0.990 | **<0.001** |
|  | Partner’s perceived partner regulation (VCR; Sum of q4a and q4bReverse) | 1.000 | 0.001 | 0.998 – 1.002 | 0.771 |
| 3.b1 | Actor decision | 1.513 | 0.001 | 1.510 – 1.516 | **<0.001** |
|  | Partner decision | 2.209 | 0.001 | 2.205 – 2.213 | **<0.001** |
|  | Actor positive regulation (VCR; Q2a) | 1.012 | 0.001 | 1.010 – 1.014 | **<0.001** |
|  | Partner positive regulation (VCR; Q2a) | 0.985 | 0.001 | 0.983 – 0.987 | **<0.001** |
|  | Actor negative regulation (VCR; Q2b) | 1.001 | 0.001 | 0.999 – 1.003 | 0.321 |
|  | Partner negative regulation (VCR; Q2b) | 1.004 | 0.001 | 1.002 – 1.006 | **<0.001** |
| 3.b2 | Actor decision | 1.514 | 0.262 | 0.906 – 2.530 | 0.114 |
|  | Partner decision | 2.235 | 0.253 | 1.363 – 3.667 | **0.001** |
|  | Actor’s perceived partner positive regulation (VCR; q4a) | 0.971 | 0.009 | 0.954 – 0.988 | **0.001** |
|  | Partner’s perceived partner positive regulation (VCR; q4a) | 1.011 | 0.009 | 0.994 – 1.029 | 0.213 |
|  | Actor’s perceived partner negative regulation (VCR; q4b) | 0.992 | 0.01 | 0.973 – 1.012 | 0.422 |
|  | Partner’s perceived partner negative regulation (VCR; q4b) | 1.012 | 0.01 | 0.993 – 1.032 | 0.228 |
| 5 | Actor decision | 1.357 | 0.249 | 0.833 – 2.212 | 0.221 |
|  | Partner decision | 2.569 | 0.243 | 1.597 – 4.133 | **<0.001** |
|  | Actor reward smile | 0.994 | 0.006 | 0.982 – 1.006 | 0.323 |
|  | Actor mouth-based appeasement | 0.991 | 0.01 | 0.971 – 1.011 | 0.378 |
|  | Actor disapproval | 0.983 | 0.03 | 0.926 – 1.043 | 0.570 |
|  | Partner reward smile | 0.998 | 0.006 | 0.986 – 1.010 | 0.740 |
|  | Partner mouth-based appeasement | 0.996 | 0.01 | 0.977 – 1.016 | 0.700 |
|  | Partner disapproval | 1.009 | 0.031 | 0.950 – 1.072 | 0.771 |

*Note*. Statistically significant (α = .05) p-values are marked in bold; AC = Automated Coding; VCR = Video Cued Recall; Q = Question, from post task questionnaire measures (see S2); see S3 for model information; Reward smile, Mouth-based appeasement, and disapproval expressions derived from Robertson et al., (*in prep*).

## **Results of logistic mixed-effect models**

*Table S7*. Results of logistic mixed-effect models, predicting participant’s decision to defect from their previous round decision to cooperate or defect and the discrepancy (between partner and self) ratings.

| Model | Lagged predictors | Odd ratios | SE | 95% CI | *p* |
| --- | --- | --- | --- | --- | --- |
| 4.a | Actor decision | 2.176 | 0.303 | 1.202 – 3.941 | **0.010** |
|  | Partner decision | 5.359 | 0.245 | 3.317 – 8.660 | **<0.001** |
|  | Discrepancy Actor Valence | 0.996 | 0.006 | 0.985 – 1.007 | 0.493 |
| 4.b | Actor decision | 2.178 | 0.303 | 1.204 – 3.942 | **0.010** |
|  | Partner decision | 5.279 | 0.246 | 3.261 – 8.547 | **<0.001** |
|  | Discrepancy Actor positive regulation | 0.998 | 0.006 | 0.985 – 1.011 | 0.735 |
| 4.c | Actor decision | 2.183 | 0.301 | 1.211 – 3.935 | **0.009** |
|  | Partner decision | 5.352 | 0.244 | 3.316 – 8.639 | **<0.001** |
|  | Discrepancy Actor negative regulation | 0.992 | 0.007 | 0.978 – 1.007 | 0.293 |
| 4.d | Actor decision | 2.199 | 0.302 | 1.217 – 3.974 | **0.009** |
|  | Partner decision | 5.354 | 0.245 | 3.313 – 8.652 | **<0.001** |
|  | Discrepancy Actor regulation | 1.002 | 0.005 | 0.993 – 1.011 | 0.678 |

*Note*. Statistically significant (α = .05) p-values are marked in bold; see S3 for model information

*Table S8*. Results of the over-time (longitudinal) APIM, predicting participant’s decision to defect from their previous round decision to cooperate or defect. Results also include the lagged values of video ratings of expression valence, and likelihood score of expressed smile from facial muscle movement.

| Model | Lagged predictors | Odd ratios | SE | 95% CI | *p* |
| --- | --- | --- | --- | --- | --- |
| 6 | Actor decision | 1.561 | 0.255 | 0.947 – 2.573 | 0.081 |
|  | Partner decision | 2.318 | 0.247 | 1.428 – 3.761 | **0.001** |
|  | Actor Duchenne smile | 0.993 | 0.005 | 0.984 – 1.002 | 0.110 |
|  | Partner Duchenne smile | 1.000 | 0.005 | 0.991 – 1.009 | 0.976 |
| 7 | Actor decision | 1.561 | 0.255 | 0.947 – 2.573 | 0.081 |
|  | Partner decision | 2.318 | 0.247 | 1.428 – 3.761 | **0.001** |
|  | Actor Duchenne smile | 0.993 | 0.005 | 0.984 – 1.002 | 0.110 |
|  | Partner Duchenne smile | 1.000 | 0.005 | 0.991 – 1.009 | 0.976 |
|  | Actor positive regulation (VCR; Q2a) | 1.014 | 0.008 | 0.998 – 1.030 | 0.091 |
|  | Partner positive regulation (VCR; Q2a) | 0.985 | 0.008 | 0.969 – 1.001 | 0.065 |

*Note*. Statistically significant (α = .05) p-values are marked in bold; see S3 for model information.

**S6. Sensitivity power analyses**

To conduct the sensitivity power analysis, we used the simulation-based approach described in Lafit et al., 2022^[[2]](#footnote-2)^. The results of the sensitivity power analysis indicated that for a design with 45 dyads and 10 rounds to yield a statistical power equal to or higher than 90%, the size for the actor effects (i.e., the effect of the participants’ own iMotions valence scores on the cooperation decision the next round) expressed in log-ratios of iMotions valence scores should be smaller than 0.95.

For full code and output, please see <https://osf.io/j59ne/>

The model parameters were as follows:

N.dyad = 45

T.obs = 10 # Number of repeated measurement occasions for each individual

c = -1.98 # Fixed intercept

a1 = 0.29 # Fixed actor effect lagged cooperation

a2 = -0.05 # Fixed actor effect of Level 1 continuous predictor

p1 = 0.94 # Fixed partner effect lagged cooperation

p2 = -0.01 # Fixed partner effect of Level 1 continuous predictor

sigma.nu = 1.67 # Std. deviation of the random intercept

mu.X = 17.58 # Mean of the Level 1 continuous predictor X

sigma.X = 32.8 # Standard deviation of the Level 1 continuous predictor X

alpha = 0.05 # Significant level

We obtained the computed power for the fixed actor effect of interest (lagged effect of the predictor):

| *Table S9*. Results of sensitivity power analysis for the fixed actor effect of interest (lagged effect of the predictor). | | |
| --- | --- | --- |
|  | a2 | Power |
| a2 | 0.9512294 | 0.948 |

1. For details of these expression scores, please see <https://osf.io/v76ha/> [↑](#footnote-ref-1)
2. Lafit, G., Sels, L., Adolf, J. K., Loeys, T., & Ceulemans, E. (2022). PowerLAPIM: An application to conduct power analysis for linear and quadratic longitudinal actor–partner interdependence models in intensive longitudinal dyadic designs. *Journal of Social and Personal Relationships*, *39*(10), 3085–3115. <https://doi.org/10.1177/02654075221080128> [↑](#footnote-ref-2)
